# Newly qualified doctors' views about whether their medical school had trained them well: questionnaire surveys

**DOI:** 10.1186/1472-6920-7-38

**Published:** 2007-10-18

**Authors:** Judith Cave, Michael Goldacre, Trevor Lambert, Kath Woolf, Alison Jones, Jane Dacre

**Affiliations:** 1 Academic Centre for Medical Education, Royal Free and University College Medical School, London, UK; 2 UK Medical Careers Research Group, Unit of Health-Care Epidemiology, Department of Public Health, Oxford University, Oxford, UK; 3 Department of Medical Oncology, Royal Free Hospital, London, UK

## Abstract

**Background:**

A survey of newly qualified doctors in the UK in 2000/2001 found that 42% of them felt unprepared for their first year of employment in clinical posts. We report on how UK qualifiers' preparedness has changed since then, and on the impact of course changes upon preparedness.

**Methods:**

Postal questionnaires were sent to all doctors who qualified from UK medical schools, in their first year of clinical work, in 2003 (n = 4257) and 2005 (n = 4784); and findings were compared with those in 2000/2001 (n = 5330). The response rates were 67% in 2000/2001, 65% in 2003, and 43% in 2005. The outcome measure was the percentage of doctors agreeing with the statement "My experience at medical school has prepared me well for the jobs I have undertaken so far".

**Results:**

In the 2000/2001 survey 36.3% strongly agreed or agreed with the statement, as did 50.3% in the 2003 survey and 58.5% in 2005 (chi-squared test for linear trend: χ^2 ^= 259.5; df = 1; p < 0.001). Substantial variation in preparedness between doctors from different medical schools, reported in the first survey, was still present in 2003 and 2005. Between 1998 and 2006 all UK medical schools updated their courses. Within each cohort a significantly higher percentage of the respondents from schools with updated courses felt well prepared.

**Conclusion:**

UK medical schools are now training doctors who feel better prepared for work than in the past. Some of the improvement may be attributable to curricular change.

## Background

A survey of newly qualified doctors from all medical schools in the UK, who undertook their first year of clinical work in 2000/2001 found that 42% of them felt unprepared by their medical school for their first clinical posts [1]. The survey also found significant and substantial differences between medical schools in how well prepared their graduates felt.

There is no consensus on how to train students to be good doctors or on how to select medical students who will make good doctors [2,3]. There is a clear consensus however, emphasised by the General Medical Council (GMC) in its document on undergraduate training entitled *Tomorrow's Doctors*, that medical schools should improve their preparation of students for their first year of working life. *Tomorrow's Doctors *states that *'students must be properly prepared for their first day as a Pre Registration House Officer*'[4]. It has also been suggested that there should be a national licensing programme to ensure all graduates are *'fit for purpose*' [5]. Following the publication of the first edition of *Tomorrow's Doctors *in 1993 [4], all UK medical schools initiated major curricular changes to bring their courses into line with the recommendations. The recommendations include not only a greater attention to preparedness to practice, but also adherence to modern educational theory, focus on attitudes, and integration of basic and clinical sciences [4]. The extent of changes necessary varied between medical schools, but all schools underwent major curricular revisions and changes to student assessment practices, with some introducing for example problem based learning. The GMC visited all medical schools to advise on the changes and to monitor progress.

Lack of preparedness has been linked to stress in junior doctors [6], and it is therefore important to investigate what might help junior doctors feel better prepared. A cohort study in Manchester found that graduates from a new problem-based learning course felt better prepared than graduates from the traditional course for 12 out of 19 of the competencies required of a newly qualified doctor [7]. However, it is not known whether there have been improvements at other medical schools in how well junior doctors feel they have been prepared for their first year of clinical work.

Our aim in this study is to report on the views of newly qualified doctors in 2003 and 2005, compared with those in 2000/2001, about their preparation for their first year of clinical work. The purpose is to begin to investigate whether the increased attention to preparedness for practice, manifested through curricular changes at UK medical schools, has resulted in improvements in the way newly qualified doctors feel.

## Methods

### Participants and questionnaires

Questionnaires asking about preparedness were sent to newly qualified doctors in the UK in 2000, 2001, 2003 and 2005. The questionnaires were sent to doctors approximately 9 months after their graduation. We have grouped the 2000 and 2001 cohorts, which included all newly qualified doctors in 2000 and a random 25% sample of those in 2001, together. The 2003 and 2005 cohorts included all graduates from those two years. All questionnaires included the statement *'My experience at medical school has prepared me well for the jobs I have undertaken so far*', presented in the same format in each survey. Respondents were invited to state their level of agreement with the statement on a five-point scale from 'strongly agree' to 'strongly disagree'.

The surveys performed in 2000/2001 and 2003 formed part of an ongoing programme of work, by the UK Medical Careers Research Group (UKMCRG) in Oxford, to establish doctors' career choices and progression. The 2005 survey formed part of a separate study of newly qualified doctors and their preparedness for looking after cancer patients funded by Cancer Research UK and based at University College London. All questionnaires were accompanied by covering letters explaining the purpose of the studies and that respondents' replies would be confidential, held securely and separately from any information that would identify them individually, and available to senior staff in the research teams only. Return of the questionnaire was considered to represent informed consent.

### Administration of the questionnaires

In 2000, 2001 and 2003, questionnaires were posted directly to the doctors' registered addresses, obtained from the GMC register. Up to four reminders were sent to non-responders. The methods have been reported previously [1]. In 2005 the GMC was unable to provide doctors' addresses so the survey was administered through 21 postgraduate deaneries as follows. Four deaneries posted the questionnaires directly to the doctors. In the other 17 deaneries the questionnaires were posted to the hospital postgraduate education centre administrators who distributed them. Up to two reminders were sent to non-responders. One deanery and eight postgraduate centres declined to participate in the 2005 questionnaire because they had previously committed to distribute deanery surveys or foundation year pilot surveys.

### Course changes

We obtained the date of course changes from the GMC website, which was set up to make available the results of the GMC monitoring of the progress of curricular change in response to *Tomorrow's Doctors*.

### Ethics

Ethical approval for the UKMCRG cohort studies and the 2005 study has been obtained through the Central Office for Research Ethics Committees (COREC).

## Results

Questionnaires were sent to 5330 doctors in 2000/2001, 4257 doctors in 2003, and 4784 doctors in 2005. The response rates were 67%, 65%, and 43% respectively (the denominator for the 2005 survey excludes doctors covered by the deanery and postgraduate centres that declined to participate). In the 2005 survey, graduates of the medical school within the deanery that declined participation were under-represented. There were no other significant differences in response rate by region or method of distribution, but female doctors were significantly more likely to respond (p < 0.001).

The results from the 2003 and 2005 surveys were compared to those from the 2000/2001 survey. In each successive cohort, an increasing proportion of doctors agreed with the statement *'My experience at medical school prepared me well for the jobs I have undertaken so far'*. In the 2000/2001 cohort, 36.3% strongly agreed or agreed, in the 2003 cohort, the corresponding percentage was 50.3% and in the 2005 cohort, it was 58.5% (chi-squared test for linear trend: χ^2 ^= 259.5, df = 1, p < 0.001; see Table 1).

**Table 1:** Improvement in preparedness over time: percentage responses to the statement that "My experience at medical school prepared me well for the jobs I have undertaken so far"

Year of survey	% who agree or strongly agree	% who neither agree or disagree	% who disagree or strongly disagree
2000/2001	36.2 (n = 1111)	22.5 (n = 689)	41.3 (n = 1262)
2003	50.3 (n = 1382)	18.9 (n = 519)	30.8 (n = 844)
2005	58.5 (n = 1195)	26.1 (n = 533)	15.3 (n = 308)

**Figure 1:**
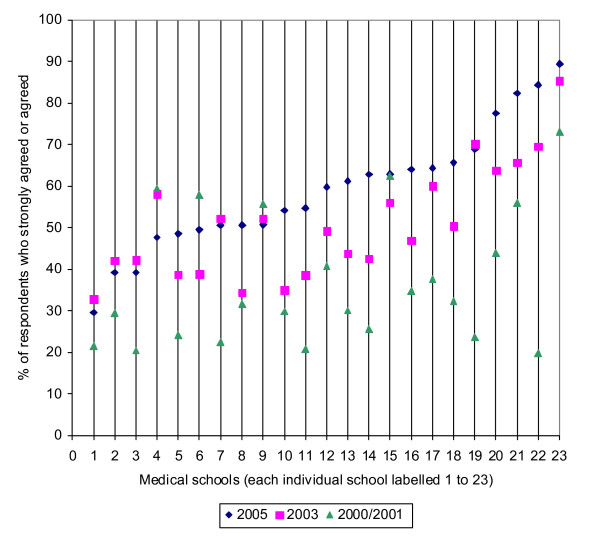
Hi-lo graph showing % of respondents who felt prepared at each school, in each cohort. The medical schools are ranked by the % of respondents who felt prepared from the 2005 survey.

Substantial and significant variation in preparedness between doctors from different medical schools, which was originally reported in the 2000/2001 study [1], was still present in the responses from 2003 and 2005. Figure 1 shows the percentages of graduates from each medical school who agreed or strongly agreed that they were well prepared, by cohort. From this figure it can be seen that in the 2003 cohort the percentage of graduates who felt well prepared ranged from 33% to 85% by medical school (pink squares), and in the 2005 cohort, the percentage of graduates who felt well prepared ranged from 30% to 89% (blue diamonds). Figure 1 also illustrates the improvements in preparedness between 2000 and 2005: it can be seen that since 2000/2001, preparedness has increased in 19 schools; dropped in three schools (numbers 4, 6 and 9); and stayed stable in one school (number 15).

Between 1998 and 2006, all UK medical schools implemented updated or 'new' courses (where implementation is defined as meaning that the majority of graduating doctors had undergone the new course) [4]. The years in which the medical schools changed their courses are shown in Table 2. At two medical schools (shown in the last row of Table 2) the new courses were gradually implemented over a number of years.

**Table 2:** Timing of introduction of new courses

Years when the new courses became fully implemented	Number of medical schools
Before 1999	7
Between 1999 and 2002	8
Between 2002 and 2004	4
After 2004	2
Ongoing over several years	2

**Figure 2:**
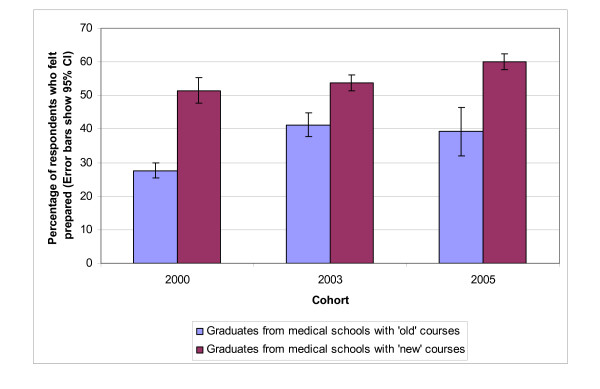
**Comparison of schools with new and old courses**. Figure 2 excludes the respondents from the two schools with ongoing course changes, and the 679 respondents from 2001 (because they only represent 25% of the cohort). To see how many schools each error bar represents, compare figure 2 and table 2 as follows. In the 2000 cohort, there were 7 medical schools with new courses and 14 with old courses (8 + 4 + 2). In the 2003 cohort there were 15 medical schools with new courses (7 + 8) and 6 with old courses (4 + 2). In the 2005 cohort there were 19 schools with new courses (7 + 8 + 4) and 2 with old courses.

Within each cohort, there were respondents from schools with new courses and respondents from schools with unchanged or 'old' courses. Figure 2 shows that, within each cohort, a statistically significantly higher percentage of the respondents from schools with new courses felt well prepared.

12 medical schools implemented new courses between 1999 and 2004 (8 between 1999 and 2002 and 4 between 2002 and 2004 – see Table 2). Surveys were therefore undertaken of graduates of both the new and the old courses run by these 12 schools. Table 3 shows that for the schools which changed their course between 1999 and 2002, there was a pronounced increase in preparedness between the 2000 and 2003 surveys (increase 28.1%; 95% CI 23.7 – 32.4) and a smaller increase between the 2003 and 2005 surveys (increase 9.2%; 95% CI 4.8–13.8%). For the four schools that changed course between 2002 and 2004 the picture is less clear: there was 15.3% increase between the 2000 and 2003 surveys (95% CI 9.5 – 21.1) and a 14.7% increase between the 2003 and 2005 surveys (95% CI 7.9 – 21.6). In these analyses we excluded the 2001 respondents (n = 679).

**Table 3:** Changes in preparedness when new courses were introduced: percentages strongly agreeing or agreeing that their medical school prepared them well

	% of respondents agreeing or strongly agreeing that their medical school prepared them well		
	
	2000 cohort	2003 cohort	2005 cohort
Schools that changed their course between 1999 and 2002 (n = 8)	29.1% (250/859)	57.2% (563/985)	66.4% (549/826)
Schools that changed their course between 2002 and 2004 (n = 4)	25.4% (119/468)	40.7% (207/508)	55.4% (187/337)

## Discussion and conclusion

The fact that such a high percentage of the newly qualified doctors in 2000/2001 did not feel well prepared for their first year of medical work was a concern. The results from the more recent qualifiers are reassuring for two reasons. First, they show that preparedness has improved significantly. Second, they suggest that changes in medical school courses may be partly responsible for the improvements. There is evidence from qualitative studies of junior doctors that certain aspects of modernised courses, for example periods of shadowing, are related to improvements in preparedness [8].

There were also improvements in preparedness in the absence of course change, suggesting other factors also affected preparedness. There are many changes which may have resulted in increased preparedness, including: increased attention by teachers to preparedness for practice; changes in student selection; and factors related to improvements in junior doctors' working environments including reduction in hours and increased supervision [9]. The changing demographics of medical school leavers, particularly the increase in graduate entrants, may also have affected the results, since graduate entrants have previously been shown to feel better prepared [10]. In the future it would be interesting to try to quantify the relative effects of these various factors upon preparedness.

The results of this study show a greater impact upon preparedness of course changes between 1999–2002 than between 2002–2004. Schools which changed their courses later appear to have experienced less benefit. There are a number of possible reasons for this. Late-changing schools may have taken measures to improve preparedness prior to their full-scale course change, for example by introducing a programme of shadowing. There could also have been methodological reasons for this difference. For the 2005 survey, the less intensive follow-up of non-responders and the indirect distribution of questionnaires via postgraduate deaneries and hospital administrators almost certainly had an adverse effect on the level of response. The first two surveys were linked to career surveys, while the 2005 survey was linked to a survey about caring for cancer patients. This may also have affected the response rate. These are potential limitations of the study, since the non-responders may have been self-selected, introducing bias. The effect of the missing graduates (working within the deanery which declined participation in the 2005 survey) is also not known. However, because there is a clear trend in the results evident across the surveys – improvements in 2005 compared with 2003, and in 2003 compared with 2000/2001 – we feel that the decision to compare the surveys is justified.

The major limitation to this study is the use of a subjective outcome measure. While subjective measures such as preparedness have strong face validity, there is no good evidence that those who feel more prepared are in fact more prepared. In a systematic review of studies which compared physicians' self assessments of ability with independent assessments of their ability, only 7 out of 20 studies demonstrated a positive correlation [11]. Formal independent assessments are now compulsory for junior doctors in the UK, and in the future it would be interesting to compare preparedness with scores in work place assessments.

The results of this study are encouraging, but they give cause for some continuing professional concern because, despite the improvements, in 2005 the percentage who agreed or strongly agreed that they had been well prepared was still only 59%. There was still striking variation between the responses of doctors from different medical schools which ranged from 89% who agreed or strongly agreed at the top of the ranking to only 30% at the bottom for the survey in 2005. Whether the results should cause public concern is less clear. As stated above, there is no good evidence that those who feel unprepared are in fact unprepared; and doctors' first year of medical work provides a supervised transition from medical student to fully registered medical practitioner.

This paper provides evidence that medical schools have given increasing recognition to the importance of preparing doctors for their first year of practice, and that they have implemented course changes to improve preparedness. Further studies are required to explore the relationship between subjective and objective measures of preparedness, as well as to follow up the long-term impact of the course changes. It is essential to study junior doctors' views about their training and competencies, especially during the present period of rapid evolution in the UK of both undergraduate and postgraduate medical education and training.

## Competing interests

JC, MG, TL, KW, and AJ have no conflicts of interest

JD is the Vice Dean of the Royal Free and University College Medical School.

## Authors' contributions

MG and TL were responsible for the surveys carried out in 2000, 2001 and 2003. JG, KW, AJ and JD were responsible for the survey carried out in 2005. All authors contributed to the conception, the data interpretation, drafting the article and the final approval of the article.

## Pre-publication history

The pre-publication history for this paper can be accessed here:


